# Effect of Congenital Anomalies of the Papillary Muscles on Mitral Valve Function

**DOI:** 10.1007/s40846-015-0011-1

**Published:** 2015-02-07

**Authors:** Yonghoon Rim, David D. McPherson, Hyunggun Kim

**Affiliations:** Division of Cardiovascular Medicine, Department of Internal Medicine, The University of Texas Health Science Center at Houston, 6431 Fannin St, MSB 1.246, Houston, TX 77030 USA

**Keywords:** Mitral valve, Parachute mitral valve, Parachute-like asymmetric mitral valve, Papillary muscle, Three-dimensional echocardiography, Computational simulation

## Abstract

Parachute mitral valves (PMVs) and parachute-like asymmetric mitral valves (PLAMVs) are associated with congenital anomalies of the papillary muscles. Current imaging modalities cannot provide detailed biomechanical information. This study describes computational evaluation techniques based on three-dimensional (3D) echocardiographic data to determine the biomechanical and physiologic characteristics of PMVs and PLAMVs. The closing and opening mechanics of a normal mitral valve (MV), two types of PLAMV with different degrees of asymmetry, and a true PMV were investigated. MV geometric data in a patient with a normal MV was acquired from 3D echocardiography. The pathologic MVs were modeled by altering the configuration of the papillary muscles in the normal MV model. Dynamic finite element simulations of the normal MV, PLAMVs, and true PMV were performed. There was a strong correlation between the reduction of mitral orifice size and the degree of asymmetry of the papillary muscle location. The PLAMVs demonstrated decreased leaflet coaptation and tenting height. The true PMV revealed severely wrinkled leaflet deformation and narrowed interchordal spaces, leading to uneven leaflet coaptation. There were considerable decreases in leaflet coaptation and abnormal leaflet deformation corresponding to the anomalous location of the papillary muscle tips. This computational MV evaluation strategy provides a powerful tool to better understand biomechanical and pathophysiologic MV abnormalities.

## Introduction

Parachute mitral valves (PMVs) and parachute-like asymmetric mitral valves (PLAMVs) are associated with congenital anomalies of the papillary muscles involving abnormal anatomy of the papillary muscles and chordae tendineae [1, 2]. PMVs have unifocal attachment of the chordae tendineae on a single papillary muscle, whereas two separate papillary muscles with a severely unequal distribution of the chordae tendineae are involved in PLAMVs [1, 3]. The chordae tendineae in PMVs and PLAMVs are often underdeveloped and deteriorate the mobility of the mitral valve (MV) leaflets due to the markedly reduced interchordal spaces [4]. This anomaly of the papillary muscle location and the chordal distribution is often accompanied by reduced size of the mitral orifice during the diastolic phase, leading to obstruction of the inflow of blood from the left atrium to the left ventricle and affecting MV function. When the chordae tendineae are excessively elongated in PMVs or PLAMVs, mitral regurgitation often occurs due to incomplete leaflet coaptation [4].

Clinical studies have demonstrated functional and anatomic characteristics of the abnormalities of PMVs and PLAMVs using echocardiography and autopsy data as well as surgical outcomes [1–4]. However, current imaging modalities and autopsy studies cannot provide detailed information pertaining to the biomechanical characteristics of PMVs and PLAMVs during valve function, which has a close relationship with abnormal blood flow due to incomplete leaflet coaptation and the degree of mitral stenosis [1, 4]. Biomechanical information about the PMV and PLAMV apparatus can help us better understand the complex developmental anomalies associated with other congenital heart diseases, and the functional and physiologic mechanism of mitral insufficiencies in PMVs and PLAMVs.

Localized mechanical stress concentration and large flexural deformation are closely related to tissue degeneration, calcification, and failure of heart valves [5–7]. Computational studies using finite element (FE) methods have demonstrated valuable additive biomechanical information pertaining to heart valve function [8–15].

In the present study, computational evaluation techniques based on three-dimensional (3D) transesophageal echocardiographic (TEE) data was utilized to determine the biomechanical characteristics of PMVs and PLAMVs over the cardiac cycle. The objectives of this study are to evaluate the closing and opening mechanics of a normal MV, two types of PLAMV with different degrees of asymmetry, and a true PMV.

## Materials and Methods

### MV Modeling Using Patient 3D TEE Data

**Fig. 1:**
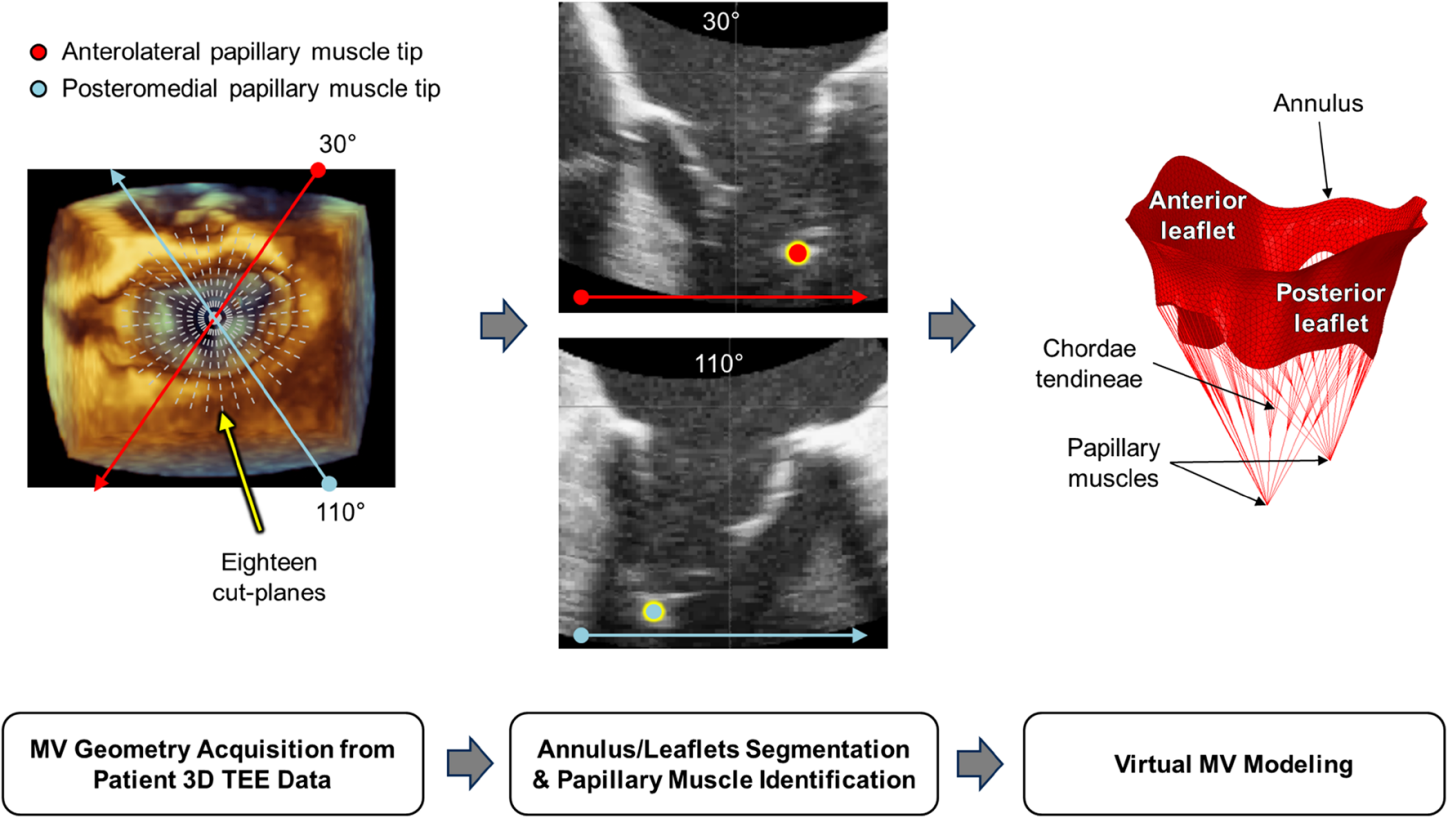
Virtual MV modeling protocol that uses patient 3D TEE data for computational FE evaluation of MV function

MV geometric data, including the anterior and posterior leaflets, annulus, and papillary muscles, in a patient with normal MV were acquired utilizing an iE33 ultrasound imaging system (Philips Medical Systems, Bothell, WA) with a 3D TEE transducer (frame rate = 25–56 fps). This computational MV evaluation study was approved by the Committee for the Protection of Human Subjects at The University of Texas Health Science Center at Houston. Recently, novel patient-specific virtual MV modeling techniques and computational MV evaluation protocols have been developed [13, 14, 16, 17]. Briefly, the mitral annulus and anterior/posterior leaflets at end diastole were segmented and traced in eighteen evenly positioned cut-plane images in the cylindrical coordinate system using a custom-designed semi-automated image processing algorithm developed in MATLAB (The Mathworks Inc., Natick, MA). Anterolateral and posteromedial papillary muscles were identified in the cut-plane images at 30° and 110° (Fig. 1). The 3D geometric data of the MV apparatus was transformed into the Cartesian coordinate for structural modeling. The mitral annulus and leaflets were created using the non-uniform rational B-spline (NURBS) surface modeling technique, meshed with 4,580 triangular shell elements, and imported into ABAQUS (SIMULIA, Providence, RI). A total of 24 marginal and 2 strut chordae tendineae were added to the MV apparatus model using 767 discretized line elements between the papillary muscles and anterior/posterior leaflets [12, 15, 18–22]. Electrocardiogram (ECG)-gated time-varying dynamic annular motion was incorporated with the virtual MV model to perform dynamic simulation of MV function over the complete cardiac cycle [13, 14]. Dynamic deformation of the papillary muscle tips was modeled by maintaining a constant distance from the top of the annulus during dynamic annular motion [23, 24].

### Modeling of the Normal MV, PLAMVs, and True PMV

**Fig. 2:**
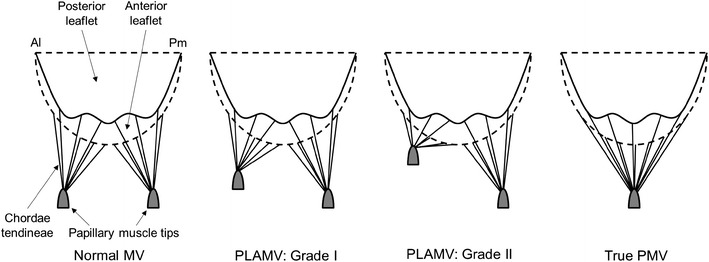
Schematic drawings for normal MV, two types of PLAMV with different degrees of asymmetry, and true PMV (*Al* anterolateral, *Pm* posteromedial)

Figure 2 demonstrates the schematic drawings of the normal MV, PLAMVs, and true PMV categorized by the degree of asymmetry of the papillary muscles. The pathologic MVs were modeled by altering the configuration of the papillary muscles in the normal MV model. Grade I and II PLAMVs were modeled with abnormally diminished anterolateral papillary muscle by displacements of 20 and 40 % from the normal papillary muscle location, respectively [2]. Therefore, the abnormal papillary muscle in the grade I and II PLAMVs was located closer to the mitral annulus and the leaflets, attached with shorter chordae tendineae compared to those for the normal MV. The true PMV was defined as a single papillary muscle to which all the chordae tendineae were focalized [4]. The focalized papillary muscle was located at the midpoint between the anterolateral and posteromedial papillary muscles of the normal MV.

### Computational Simulation of MV Function

The MV leaflets and chordae tendineae were modeled as nonlinear hyperelastic materials incorporating previously published stress–strain data [12, 25]. The thicknesses of the anterior and posterior leaflets were set to 1.31 and 1.26 mm, respectively [26]. Cross-sectional areas of the strut chordae and the anterior and posterior marginal chordae were 0.61, 0.29, and 0.27 mm^2^, respectively [12]. The density and Poisson’s ratio of the whole MV apparatus were set to 1,100 and 0.48 kg/m^3^, respectively [9, 10, 15]. Coaptation between the two leaflets was modeled using the general contact algorithm with the penalty method [14]. The friction coefficient was 0.05 [27]. A time-varying physiological transvalvular pressure gradient was applied on the leaflets across the cardiac cycle for dynamic computational MV evaluation (maximum systolic pressure = 13.4 kPa, maximum diastolic pressure = −0.3 kPa) [13, 14]. Further details of the protocol for MV modeling and dynamic FE analysis are described in our previous studies [13, 14].

### Geometric Evaluation of the Normal MV, PLAMVs, and True PMV

**Fig. 3:**
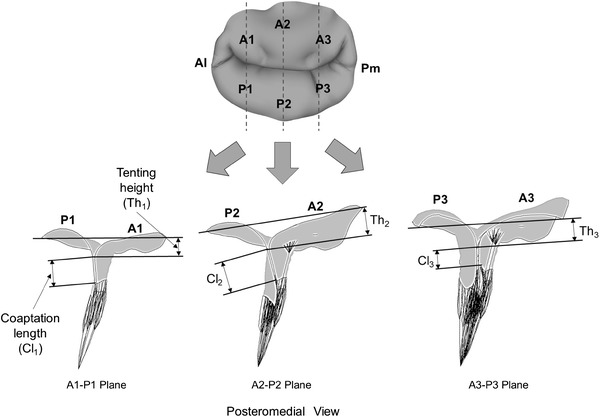
Three anteroposterior planes for assessing geometric parameters (tenting height and coaptation length) of the MVs at peak systole (*A* anterior, *P* posterior, *Cl* coaptation length, *Th* tenting height)

Several parameters can be used to define the degree of leaflet tethering such as tenting height, leaflet angle, and coaptation length [28, 29]. Morphologic evaluation of the MV configuration at peak systole was performed by determining two geometric parameters; tenting height and coaptation length. Three anteroposterior cut-planes (A1–P1, A2–P2, A3–P3) were identified to assess the geometric parameters in each MV model (Fig. 3).

The tenting height was defined by measuring the distance between the top of leaflet coaptation and the mitral annulus plane (i.e., the plane fitted to the annular curvature points by the least-squares method). The coaptation length was defined as the distance from the free marginal leaflet edge to the top of leaflet coaptation.

In order to assess the obstruction of blood inflow from the left atrium to the left ventricle during the diastolic phase induced by the change of mitral orifice size in the PLAMVs and true PMV, the orifice-to-annulus ratio was determined by measuring the surface planar projection area of the orifice over the least-squares plane of the annulus at end diastole.

## Results

### Stress Distributions in the Normal MV, PLAMVs, and True PMV at Peak Systole

**Fig. 4:**
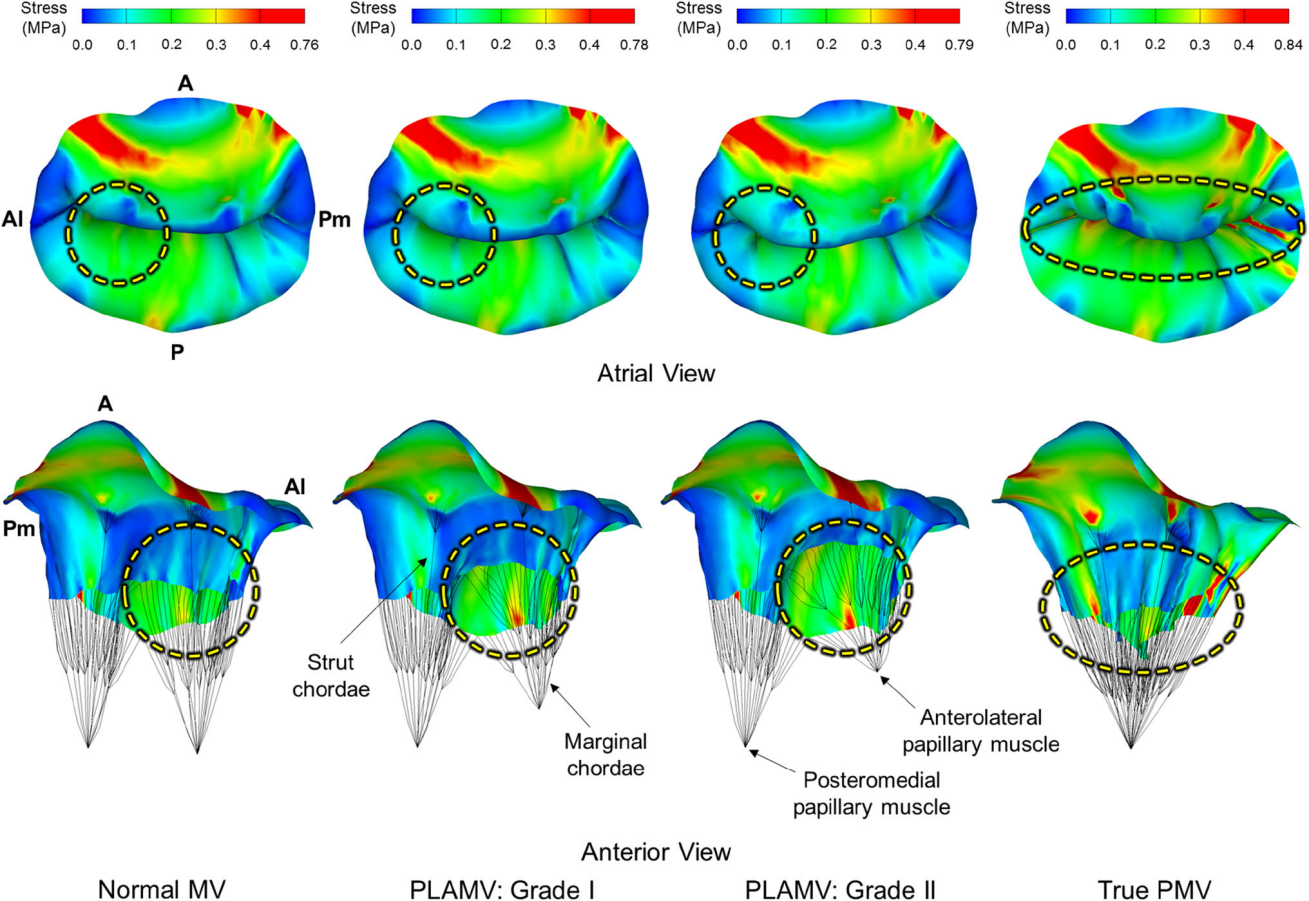
Stress distributions across the MV leaflets for the normal MV, grade I PLAMV, grade II PLAMV, and true PMV at peak systole. *Circles* indicate increased anterior leaflet bulging in the PLAMVs and severely wrinkled leaflet deformation in the true PMV

Stress distributions across the MV leaflets in the normal MV, PLAMVs, and true PMV at peak systole are displayed in Fig. 4. Large stress concentrations (>0.4 MPa) were found in the vicinity of the mitral annulus-aorta junction in the anterior leaflet along the circumferential direction (i.e., Al–Pm direction). The normal MV, grade I PLAMV, grade II PLAMV, and true PMV demonstrated comparable maximum stress values of 0.76, 0.78, 0.79, and 0.84 MPa, respectively. The posterior leaflet demonstrated large stress distributions toward the radial direction (i.e., A–P direction). The anterolateral and posteromedial commissural regions revealed relatively low stress values (<0.1 MPa). In the PLAMVs, the anomalies of the asymmetric papillary muscles produced no considerable changes in overall stress distributions across the MV leaflets. The PLAMVs demonstrated increased anterior leaflet bulging near the anterolateral commissural region in proportion to the degree of asymmetry of the papillary muscle locations. Although similar overall patterns of stress distribution were found, the true PMV demonstrated severely wrinkled leaflet deformation and densely narrowed interchordal spaces at peak systole compared to those of the normal MV. Increased large stress concentration in the commissural areas toward the radial direction was found in the true PMV.

### Mitral Orifice Sizes of the Normal MV, PLAMVs, and True PMV

**Fig. 5:**
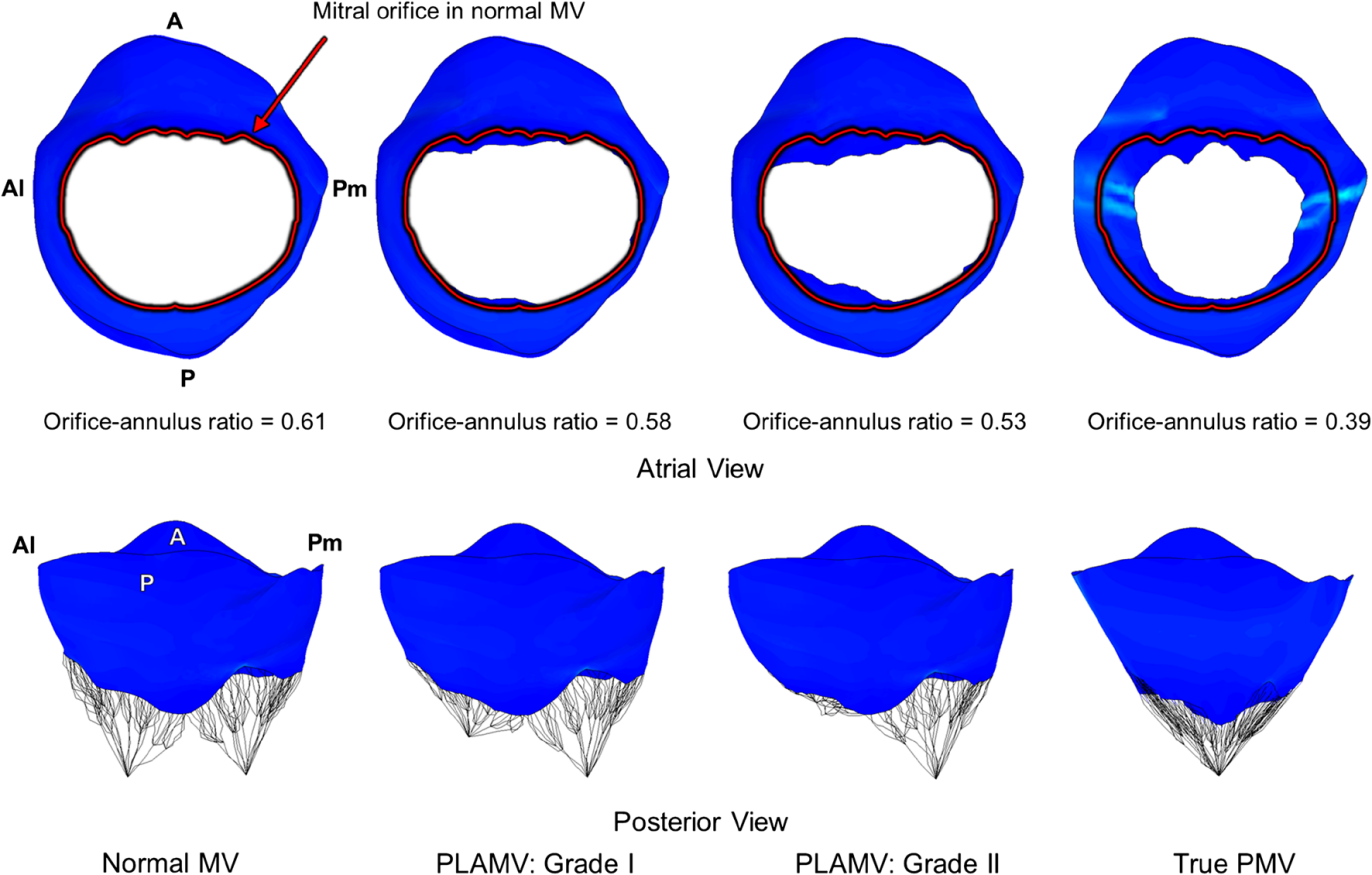
Orifice-to-annulus ratio data of the normal MV, grade I PLAMV, grade II PLAMV, and true PMV at end diastole

The orifice-to-annulus ratio of the normal MV was 0.61 at end diastole (Fig. 5). Grade I and II PLAMVs demonstrated reduced orifice-to-annulus ratios of 0.58 and 0.53, respectively. This indicates that the orifice-to-annulus ratio decreases as the degree of asymmetry of the papillary muscle locations increases due to the shortened distance between the anterolateral papillary muscle tip and the annulus. However, the anterolateral commissural region in the PLAMVs retained a proper amount of MV opening. In the true PMV, the orifice-to-annulus ratio at end diastole further decreased to 0.39, demonstrating a tapered leaflet shape (from the posterior view) with a largely reduced MV opening.

### Contact Mechanism of Leaflet Coaptation in the Normal MV, PLAMVs, and True PMV

**Fig. 6:**
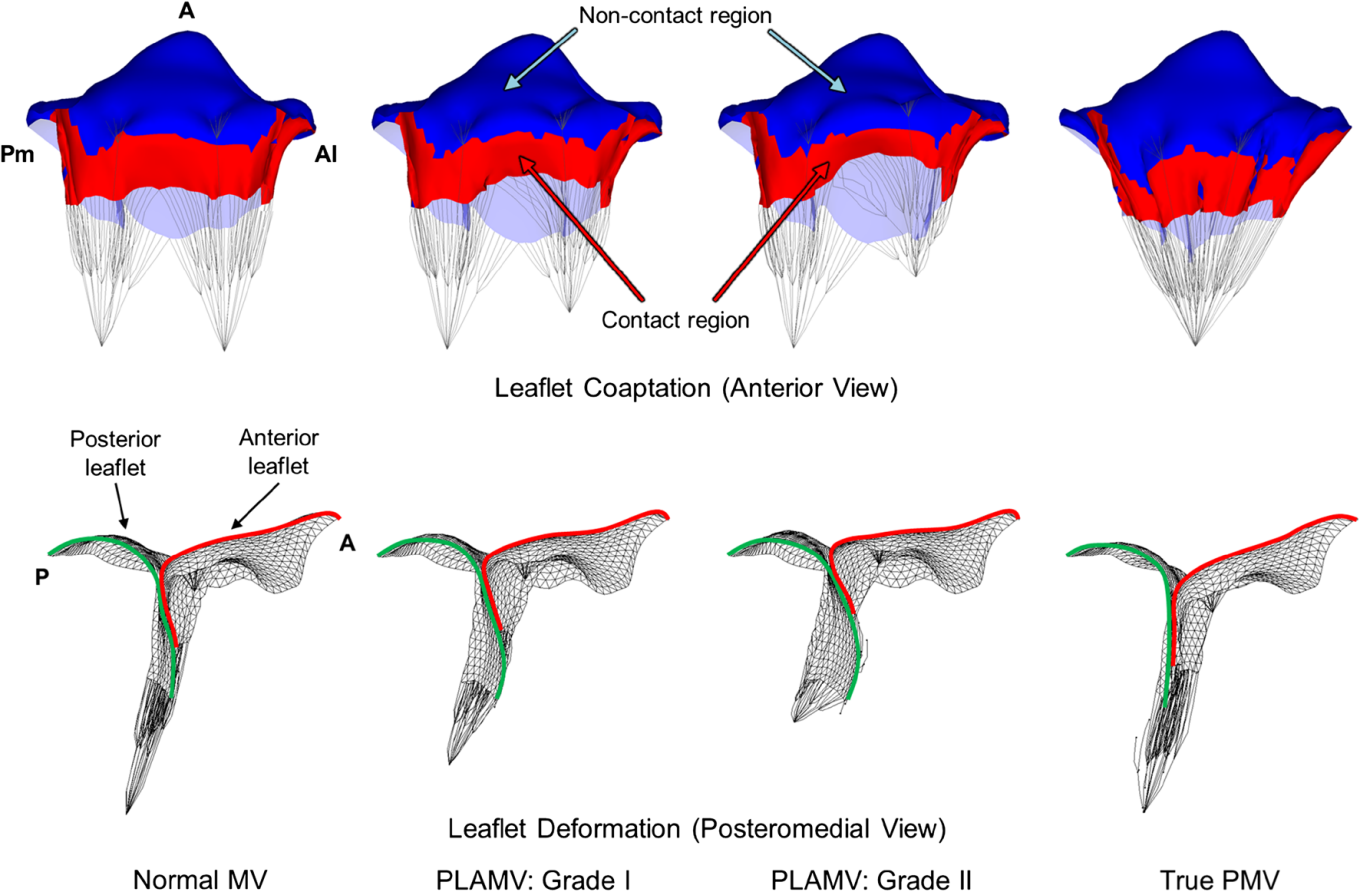
Leaflet coaptation and deformation of the normal MV, grade I PLAMV, grade II PLAMV, and true PMV

Leaflet coaptation and deformation in the normal MV, PLAMVs and true PMV at peak systole are demonstrated in Fig. 6. The normal MV showed sufficient leaflet coaptation from the posteromedial commissure to the anterolateral commissure. There was no leaflet malcoaptation region leading to mitral regurgitation or prolapse. In the PLAMVs with increased papillary muscle asymmetry, leaflet coaptation decreased in the anterolateral commissural and anterior regions compared to those for the normal MV (anterior view). The posteromedial region in both PLAMVs retained degrees of leaflet coaptation similar to that of the normal MV. With the reduced leaflet coaptation, the PLAMVs demonstrated a sharp-angled deformation in the anterior leaflet in proportion to the papillary muscle asymmetry (posteromedial view). In the true PMV, the tapered leaflet morphology revealed uneven and complex leaflet coaptation with flatter leaflet deformation in the cross-sectional view. However, the overall decrease of leaflet contact in the true PMV was not as large as that for the PLAMVs.

**Fig. 7:**
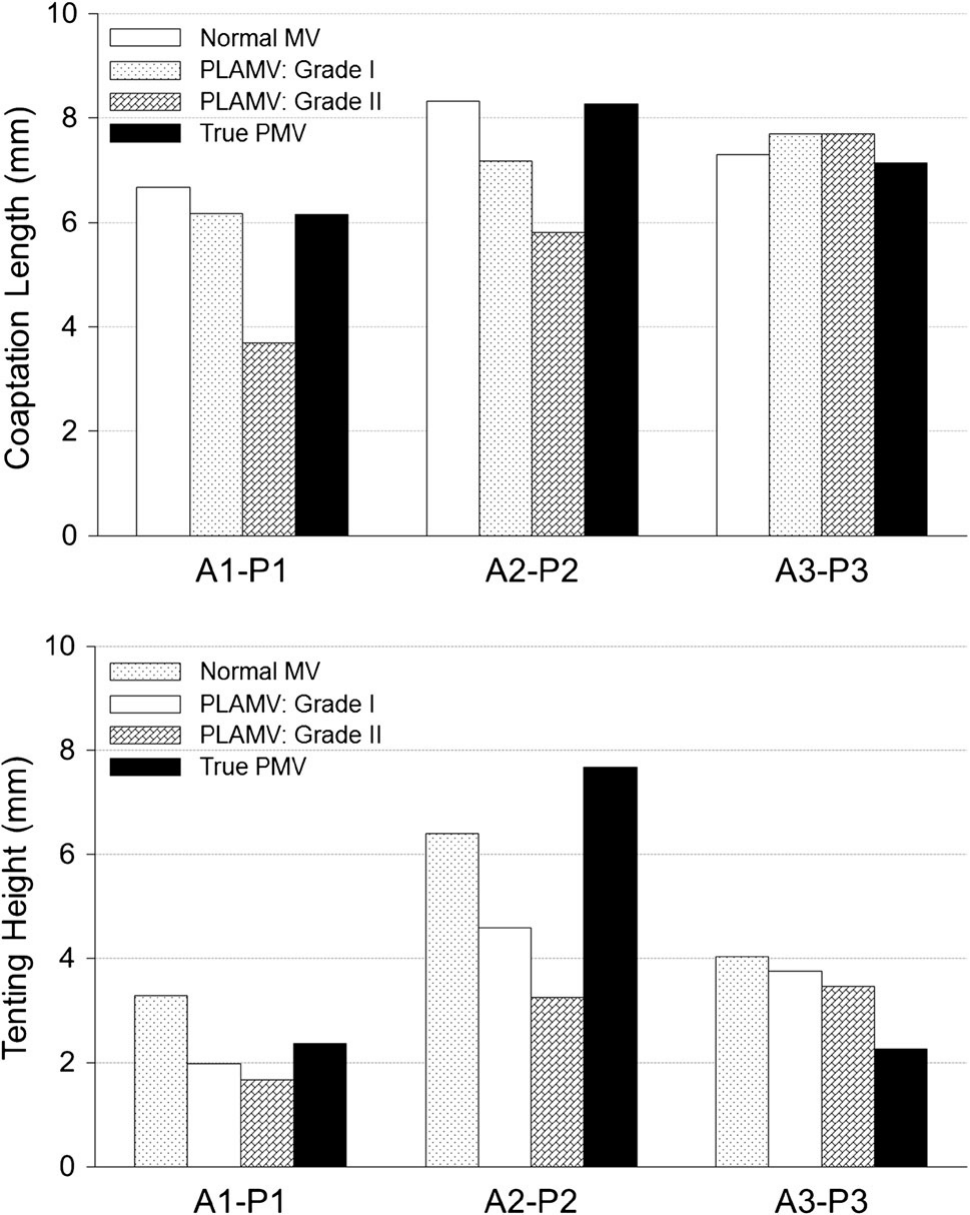
Coaptation lengths and tenting heights of the normal MV, grade I PLAMV, grade II PLAMV, and true PMV

Coaptation lengths and tenting heights in the normal MV, PLAMVs, and true PMV are compared in Fig. 7. Although the stress distributions and peak stress values were similar between these MVs, the coaptation lengths and tenting heights demonstrated a difference related to the degree of papillary muscle asymmetry. In the normal MV, the coaptation length and the tenting height in the A2–P2 plane were 8.3 and 6.4 mm, respectively. The grade I PLAMV demonstrated reduced coaptation length (7.2 mm) and tenting height (4.6 mm) in the middle of the valve (A2–P2 plane). Further decreased values (5.8 and 3.2 mm, respectively) were found in the grade II PLAMV. A similar pattern of decrease of coaptation length and tenting height was found in the A1–P1 plane of the PLAMVs. The true PMV revealed a largely reduced tenting height in the A3–P3 plane.

## Discussion

PMVs and PLAMVs are often grouped together and considered as a single lesion [1]. Only a few studies that have investigated the influence of various types of MV anomaly on the physiologic performance of these valves [1–3]. In these studies, echocardiography and autopsy databases were utilized to identify morphological and functional anomalies. Evaluation of biomechanical information of PMVs and PLAMVs can help us to better understand the pathophysiologic characteristics of these valves. However, no studies have utilized biomechanics to investigate the functional and physiologic mechanism of these pathologic MVs, in particular the effect of anomalous papillary muscles on abnormal functional characteristics of PMVs and PLAMVs.

In this study, computational modeling and simulation techniques based on 3D echocardiographic data were used to quantitatively evaluate the extent and severity of abnormality and the leaflet contact mechanisms of PMVs and PLAMVs, and compare them to normal MV physiology. It was found that (a) the PLAMVs and true PMV demonstrated comparable maximum leaflet stress values to that of the normal MV, (b) there was a strong correlation between the reduction of mitral orifice size and the degree of abnormality of the papillary muscles, and c) decreased leaflet coaptation and tenting height corresponded to the degree of papillary muscle asymmetry.

A previous study demonstrated that displacement of the papillary muscle location created abnormal stresses in the valvular structure, increasing the potential for mitral regurgitation [30]. They simulated MV function, progressively moving the posteromedial papillary muscle tip outward (i.e., medially), and determined the effect of papillary muscle disposition. The present study more specifically investigated PMVs and PLAMVs, which have a variety of anatomic anomalies of the papillary muscles. The focus of the investigation was on the effect of the initial malformation of the papillary muscle anatomy categorized by the standard clinical guidelines (Fig. 2). It is important to design correct virtual MV models for PMVs and PLAMVs that incorporate congenital anomalies of asymmetric papillary muscles into the modeling protocol. Our modeling used state-of-the-art computational simulation techniques and patient-specific MV geometric data attained from 3D echocardiography.

The PLAMVs and true PMV demonstrated orifice sizes 4.9–13 and 36 % smaller than that of the normal MV (Fig. 5). Reduction of mitral orifice size demonstrated a strong correlation with the degree of papillary muscle asymmetry in the PLAMVs and the abnormal morphology with densely narrowed interchodal space in the true PMV. Reduced orifice size not only decreases inflow from the left atrium, but may induce anomalous blood flow in the left ventricle that can lead to adaptive remodeling.

Another important observation with increased asymmetry of the papillary muscles is the decreased leaflet contact area and increased leaflet bulging. In particular, the anterolateral commissural region where the anomalous papillary muscle was located in the PLAMVs clearly demonstrated these characteristics (Fig. 6). The relationship between the decreased leaflet contact and the increased leaflet bulging toward the left atrium can be explained by tensile strains and stresses in the chordae tendineae. The shortened chordae tendineae of the posterior leaflet between the belly region and the anterolateral commissural region produce large increases of chordal stresses and strains. Reduced mobility of the posterior leaflet margin due to the shortened chordal lengths induces a pushing effect toward the anterior leaflet, leading to anterior leaflet bulging.

The degree of abnormal leaflet contact and leaflet bulging in the true PMV was as considerable as those in the PLAMVs. This indicates that unifocal disposition of the papillary muscles may have little effect on the degree of leaflet contact size as long as the chordae tendineae retain symmetric motions over the cardiac cycle. However, it is likely that severely wrinkled leaflet deformation of the true PMV may alter the biomechanical and functional characteristics of the valve and its apparatus in the long term.

There are several limitations and simplifications in the present study. In order to comprehensively evaluate the functional abnormalities of PMVs and PLAMVs, it is necessary to take into consideration various pathophysiologic factors as congenital pathologies of PLAMVs and PMVs are commonly associated with other valvular anomalies. The biomechanical conditions of PMVs and PLAMVs were simplified by focusing only on the alteration of the papillary muscle positions without adding other geometric alterations involving the mitral annulus, leaflets, or chordae tendineae. To more completely evaluate the biomechanical characteristics of PLAMVs and PMVs, our studies should be extended to investigate other potential pathologic factors in PLAMVs and PMVs. It was assumed that the papillary muscle tips were at a single position. However, the methods utilized can easily incorporate other characteristics of the apparatus. There is a large variability in the morphology of PMVs and PLAMVs, and specific diagnosis and therapeutic implications for individual PMV or PLAMV patients are strongly dependent on individual valvular anatomy. The present study investigated three types of the most representative PMV and PLAMV anatomies involving congenital anomalous papillary muscles.

Although the leaflet geometry and dynamic annular motion of the normal MV were imported from 3D TEE data, the chordae tendineae were modeled based on previously reported clinical data [12, 20, 22]. Experimentally determined data of material properties, thicknesses, tissue density, Poisson’s ratio, and friction coefficients of the leaflets and chordae from previously studies were employed since patient-specific data of these quantities are not available in vivo. Rigorous quantitative and qualitative validation data for our virtual MV simulation strategy can be found in our previous studies [13, 14, 17]. A variety of types of material characteristics can be incorporated into our computational simulation protocol. Continuous upgrades of our system using more realistic human tissue characteristics whenever new data are available are planned. Dynamic FE simulations were performed with physiologic damping conditions to best mimic the physiologic effects of blood flow on the leaflets and chordae structure in an FE simulation platform. A fluid–structure interaction approach is required to fully demonstrate blood flow characteristics around the MV apparatus.

## Conclusion

A novel computational strategy was demonstrated to evaluate the effects of congenital anomalies of the papillary muscles on MV function. Our findings demonstrated that there were considerable decreases of leaflet coaptation and abnormal deformation of leaflet morphology corresponding to the anomalous location of the papillary muscle tips, resulting in reduced mitral orifice size without significant mitral regurgitation. This computational MV evaluation strategy accompanied by conventional patient 3D echocardiographic data provides a powerful tool to better understand the biomechanical and pathophysiologic abnormalities in patients with MV abnormalities. By better modeling the MV apparatus, physiologic characteristics (stenosis, regurgitation, and malcoaptation) can be identified. This would allow better follow-up of congenital MVs and allow better planning of surgical treatment approaches in patients prior to operation.
